# Association Between HLA Polymorphisms and Non-Alcoholic Fatty Liver Disease in Patients with Rheumatoid Arthritis: An Observational Study

**DOI:** 10.3390/diseases14030113

**Published:** 2026-03-22

**Authors:** Tatjana Zekić, Nataša Katalinić, Filip Blažić, Nada Starčević Čizmarević, Aleksandar Čubranić

**Affiliations:** 1 Clinical Hospital Center Rijeka, 51000 Rijeka, Croatia; athalblazic@gmail.com (F.B.); acubrani@yahoo.com (A.Č.); 2 Clinical Hospital Center Rijeka, Faculty of Medicine, University of Rijeka, 51000 Rijeka, Croatia; nkatalinic1@gmail.com; 3 Faculty of Medicine, University of Rijeka, 51000 Rijeka, Croatia; nadasc@uniri.hr

**Keywords:** HLA-DR antigens, inflammation, metabolic syndrome, non-alcoholic fatty liver disease, arthritis, rheumatoid

## Abstract

Background/Objectives: This observational study investigated associations between human leukocyte antigen (HLA) polymorphisms and imaging-defined hepatic steatosis (non-alcoholic fatty liver disease—NAFLD) and liver fibrosis in patients with rheumatoid arthritis (RA). Methods: Steatosis was assessed by transient elastography (FibroScan) and defined as controlled attenuation parameter (CAP) ≥ 275 dB/m; fibrosis was defined as liver stiffness measurement ≥ 8 kPa. We tested 11 frequent HLA alleles (HLA-A*02, HLA-B*07, HLA-B*08, HLA-B*27, HLA-B*35, HLA-B*44, HLA-B*51, HLA-DRB1*11, HLA-DRB1*14, HLA-DRB1*15, and HLA-DRB1*16). Associations were evaluated using multivariable logistic regression (individual and omnibus models) adjusted for age, body mass index (BMI), triglycerides, and glucose. Results: A total of 176 patients with rheumatoid arthritis were enrolled. NAFLD/steatosis was present in 35.2% of patients (n = 62), and fibrosis in 10.8% (n = 19). No HLA allele was significantly associated with steatosis or fibrosis after correction for multiple testing. BMI and triglycerides were independently associated with steatosis (BMI OR 1.22, 95% CI 1.12–1.34; triglycerides OR 1.48, 95% CI 1.04–2.18). For fibrosis, HLA-DRB1*15 showed the strongest trend-level association (OR ~2.6–2.9) but did not remain significant after correcting for multiple testing. Conclusions: In this RA cohort, metabolic factors (particularly BMI and triglycerides) were the dominant predictors of CAP-defined steatosis. No robust association between the tested HLA markers and steatosis or fibrosis was identified. Trend-level signals—most notably HLA-DRB1*15 for fibrosis—should be considered hypothesis-generating and warrant replication in larger, adequately powered cohorts.

## 1. Introduction

Rheumatoid arthritis (RA) is a systemic autoimmune disease strongly associated with human leukocyte antigen (HLA)-DRB1 alleles, which remain the major genetic susceptibility locus for RA [1]. RA affects approximately 0.5–1% of adults and, if inadequately treated, can lead to progressive joint destruction, deformity, and disability; it may also present with extra-articular manifestations and a substantial cardiometabolic comorbidity burden. Long-term therapy with disease-modifying antirheumatic drugs is therefore required in most patients, and hepatic safety is a practical consideration when selecting and monitoring treatment. Methotrexate (MTX) remains the anchor conventional synthetic agent in RA; although generally well tolerated, MTX has recognized hepatotoxic potential and routine follow-up (including liver biochemistry) is recommended in clinical practice. In contrast, biologic and targeted synthetic DMARDs are overall less strongly linked to chronic hepatotoxicity than MTX, but they can still be associated with transient elevations in liver enzymes and, rarely, more severe drug-induced liver injury.

Steatotic liver disease (SLD) encompasses hepatic steatosis identified by imaging or histology; the term metabolic dysfunction-associated steatotic liver disease (MASLD) has been introduced to emphasize the cardiometabolic context of steatotic liver disease. In much of the existing literature, this condition is referred to as non-alcoholic fatty liver disease (NAFLD). In this study, we operationalized the liver phenotype as imaging-defined steatosis based on controlled attenuation parameter (CAP) on transient elastography (CAP ≥ 275 dB/m). NAFLD is the most prevalent chronic liver condition globally, affecting approximately 25–30% of adults [2]. A recent meta-analysis reported a pooled prevalence of NAFLD in RA of 22%, and, when assessed by FibroScan, 33.3% [3]. NAFLD is strongly associated with obesity, insulin resistance, and metabolic syndrome, but its pathogenesis is multifactorial, involving genetic, epigenetic, and immunological factors [4]. Emerging evidence suggests that immunogenetic factors, including HLA polymorphisms, may influence the risk and severity of NAFLD in patients with RA [5]. Understanding these associations could provide insights into disease pathophysiology and inform personalized therapeutic strategies. HLA molecules, encoded within the major histocompatibility complex (MHC) on chromosome 6, are critical for antigen presentation and immune regulation. Their polymorphic nature has been linked to autoimmune and metabolic disorders, suggesting a plausible role in NAFLD pathophysiology [6]. While traditional risk factors, such as body mass index (BMI) and waist circumference (WC), remain the strongest predictors of NAFLD, recent evidence suggests that variation within the HLA system may influence susceptibility to NAFLD and its progression [7]. In this context, non-invasive liver assessment may complement laboratory monitoring: best-practice recommendations for optimizing MTX use and follow-up in RA have been summarized previously [8], and CAP-based transient elastography data from our group suggest that steatotic liver disease is common in RA, supporting the clinical relevance of FibroScan-based screening in this population [9].

However, data on HLA associations remain limited and heterogeneous across populations. Most studies are small-scale and lack longitudinal follow-up, underscoring the need for well-designed observational studies to clarify the role of HLA polymorphisms in the onset and progression of NAFLD. The objective of this study is to determine whether specific HLA alleles are associated with imaging-defined NAFLD (CAP-based steatosis; CAP ≥ 275 dB/m) or liver fibrosis (LSM ≥ 8 kPa) in patients with RA.

## 2. Materials and Methods

### 2.1. Study Design and Participants

A cross-sectional study was conducted at the Department of Rheumatology and Clinical Immunology, Clinical Hospital Center Rijeka (Croatia), in 2023 and 2024. Participants were recruited from the hospital’s rheumatology outpatient service. Consecutive eligible patients attending routine visits were invited to participate.

Inclusion criteria were age ≥ 18 years and fulfillment of the ACR/EULAR classification criteria for RA [10]. If participants were receiving methotrexate (MTX) at enrolment, inclusion required that the MTX dose had been stable for at least 6 months prior to enrolment. Cumulative MTX dose was calculated from weekly doses and months of treatment by hand because of dose increases and decreases over the years of treatment and variety of disease activity.

Exclusion criteria were alcohol consumption >20 g/day, viral hepatitis, autoimmune liver disease, malignancy (current or previous), thyroid disease, use of non-steroidal anti-inflammatory drugs, pregnancy, positive antinuclear antibody (ANA), and incomplete data. In addition, patients receiving systemic glucocorticoids at doses >10 mg/day prednisone equivalent were excluded to minimize confounding by high-dose steroid exposure. Physical activity was assessed by clinical history; participants reported regular physical activity (≥3 sessions/week of ≥30 min), and individuals with disability severe enough to preclude normal physical activity were not enrolled.

### 2.2. HLA Typing and Liver Assessment

HLA typing and liver imaging were used to define allele status and to determine NAFLD and fibrosis outcomes. The analytical dataset comprised 18 variables: one demographic variable (gender); two hepatic outcome measures—controlled attenuation parameter (CAP ≥ 275 dB/m) for fatty liver and liver stiffness measurement (LSM ≥ 8 kPa) for liver fibrosis [11,12]; eleven HLA markers (HLA-A*02, HLA-B*07, HLA-B*08, HLA-B*27, HLA-B*44, HLA-B*35, HLA-B*51, HLA-DRB1*11, HLA-DRB1*14, HLA-DRB1*15, and HLA-DRB1*16); and four clinical covariates (age, body mass index (BMI), triglycerides (TR; RV < 1.7 mmol/L), and glucose (RV, 4.5–6.5 mmol/L)). Diabetes status, hypertension status, and statin use, which are more specific for MASLD, were not available in a sufficiently complete and standardised manner for inclusion in the regression models; this is addressed as a limitation. Detailed bDMARD/tsDMARD subclassification was not included in the covariate set to preserve model parsimony; this choice is supported by our prior CAP-based RA study, in which treatment class was not associated with steatosis [9]. In this cohort, when we compared the occurrence of NAFLD in patients on biological therapy and those without biological therapy, there was no association between therapy and fatty liver (Fisher’s exact test, *p* = 0.6). Molecular HLA typing was performed using polymerase chain reaction–sequence-specific oligonucleotide (PCR-SSO) and polymerase chain reaction–sequence-specific primer (PCR-SSP) assays. HLA-DRB1 alleles were typed at low resolution using PCR-SSP.

Selection of HLA markers for association testing was prespecified and based on (i) availability from the clinical HLA typing panel and (ii) sufficient carrier frequency in the cohort to permit stable estimation and inspection of cell counts. Accordingly, we restricted analyses to the most prevalent alleles observed in this cohort (Table 1) and did not test rare alleles with very low carrier counts, which would be underpowered and prone to sparse-data instability.

### 2.3. Transient Elastography (FibroScan) Acquisition and Quality Criteria

Transient elastography (FibroScan; Echosens, Paris, France) was performed as described. Measurements were obtained from the right liver lobe through the intercostal spaces with participants in the supine position and the right arm maximally abducted. Probe type (M or XL) was selected according to manufacturer recommendations to ensure an appropriate measurement depth, especially in obese patients. The FibroScan software (https://www.echosens.com/products/my-fibroscan/ (accessed on 19 March 2026))automatically rejected invalid measurements. The success rate was defined as the ratio of valid measurements to the total number of attempts. Examinations were considered reliable if they included ≥10 valid measurements, a success rate ≥60%, and an interquartile range-to-median ratio (IQR/median) ≤30%; only reliable examinations were included in the analysis [13]. Examinations were performed by trained operators. The overall failure/unreliable examination rate was 3 (%), and these examinations were excluded from analysis.

### 2.4. Ethics

The study was conducted in accordance with the principles of the 1975 Declaration of Helsinki. The Ethics Committee of the Clinical Hospital Center Rijeka approved the study (approval no.: 2170-29-02/1-23-2). All participants provided written informed consent.

### 2.5. Statistical Analyses

Analyses comprised individual models and omnibus models for each outcome, using logistic regression with multiple-testing correction to identify significant associations. For the individual analyses, binary logistic regression was used to evaluate the association between each HLA marker and each outcome. Eleven separate models were fitted per outcome, each including a single HLA marker as the primary predictor. To ensure adequate model stability given the number of outcome events (*n* = 62 for NAFLD; *n* = 19 for fibrosis), a parsimonious set of four covariates was selected a priori on the basis of established clinical relevance to NAFLD and liver fibrosis risk: age (continuous, years), BMI (continuous, kg/m^2^), triglycerides (continuous, mmol/L), and glucose (continuous, mmol/L). Highly correlated anthropometric variables (weight, waist circumference, hip circumference) were excluded to reduce redundancy with BMI and to improve the events-per-variable (EPV) ratio. Cholesterol, current MTX use, and cumulative MTX dose were also excluded as they showed no significant associations in preliminary analyses. This specification yielded approximately 12 EPV for NAFLD individual models (62 events, 5 parameters) and approximately 4 EPV for fibrosis individual models (19 events, 5 parameters).

For the NAFLD outcome, individual HLA models were fitted using standard maximum-likelihood logistic regression via the glm (generalized linear model) function in R with a binomial family and logit link, as the EPV ratio was adequate (≈12). Odds ratios (ORs) with 95% confidence intervals (CIs) were derived using profile likelihood.

For the fibrosis outcome, where the low event count (n = 19) yielded an EPV of approximately 4, individual HLA models were fitted using Firth’s penalized logistic regression to mitigate small-sample bias and problems arising from sparse data or quasi-complete separation. Firth’s method was implemented via the logistf package in R (version 0.24.6). Profile penalized likelihood confidence intervals, and *p*-values are reported for all Firth models.

Then, omnibus models were fitted for each outcome, including all eleven HLA markers simultaneously along with the same four covariates (15 parameters total), to assess the joint contribution of HLA markers while controlling for potential confounding among HLA types. Given the reduced EPV ratios in the omnibus specification (approximately 4 for NAFLD with 62 events; approximately 1.3 for fibrosis with 19 events), both omnibus models were fitted using Firth’s penalized logistic regression.

To account for simultaneous testing of eleven HLA markers, the Benjamini–Hochberg (BH) procedure was applied to control the false discovery rate (FDR) within each model set (individual NAFLD, individual fibrosis, omnibus NAFLD, omnibus fibrosis). Both nominal and FDR-adjusted *p*-values are reported. Statistical significance was assessed at α = 0.05 after adjustment.

For all HLA markers, the reference category was absence of the marker (“NO”); accordingly, ORs represent the odds of the outcome among carriers relative to non-carriers. Raw 2 × 2 frequency tables (carrier status × outcome) are provided for all alleles to allow inspection of cell counts. Variance inflation factors (VIFs) were calculated for a representative model to assess the absence of problematic multicollinearity among the reduced covariate set; all VIFs were <1.2. All analyses were performed in R (version 4.3.3; R Foundation for Statistical Computing, Vienna, Austria). Graphical abstract was made in Microsoft Copilot 365.

## 3. Results

### 3.1. Non-Alcoholic Fatty Liver Disease

A total of 176 patients with rheumatoid arthritis were enrolled. Based on BMI, 66 (37.5%) of participants were overweight (25–29.9 kg/m^2^) and 40 (23.8%) were obese (≥30 kg/m^2^). Disease activity at assessment was characterized using DAS28-CRP (2.65 ± 1.1; median [1, 56]). Of them 56.8% were in remission (DAS28CRP < 2.6), 26.7% had low disease activity (DAS28CRP 2.6–3.2), 11.3% medium disease activity (DAS28CRP 3.2–5.1), and 5.1% had high disease activity (DAS28CRP > 5.1). Non-alcoholic fatty liver disease was present in 35.2% of participants (n = 62). Compared with those without NAFLD, affected patients had significantly higher weight, BMI, waist circumference (WC), and hip circumference (HC). The sample was predominantly female (n = 152, 86.4%) with 24 males (13.6%). The mean age was 59.2 years (SD = 9.3). Baseline characteristics and prevalence data of specific HLA alleles stratified by NAFLD status are shown in Table 1. Diabetes mellitus was present in 10 of 176 participants (5.6%). Current systemic glucocorticoid therapy was used by 97 participants (55%), typically at low dose (mean daily dose 5.9 mg prednisone equivalent); 18 patients received 7.5–10 mg/day (patients receiving >10 mg/day were excluded). In an exploratory analysis, current prednisone dose was not correlated with CAP (*p* = 0.10). Carrier frequencies for each HLA allele stratified by NAFLD status are presented in Table 2.

Across the eleven individual HLA models (each adjusted for age, BMI, triglycerides, and glucose), no HLA marker was significantly associated with NAFLD at either the nominal or FDR-adjusted level (Table 3). BMI was consistently the strongest predictor across all models (all *p* < 0.001). Triglycerides showed borderline-to-significant associations in most models. Among HLA markers, HLA-DRB1*16 showed the smallest nominal *p*-value (OR = 0.49, 95% CI [0.17, 1.30], *p* = 0.170, *p*_adj_ = 0.632), followed by HLA-DRB1*15 (OR = 0.53, 95% CI [0.19, 1.38], *p* = 0.208, p_adj_ = 0.632). None approached statistical significance after correction for multiple testing.

### 3.2. NAFLD Omnibus Model

As no individual HLA marker reached statistical significance, an omnibus model was fitted that included all 11 HLA markers simultaneously, adjusted for age, BMI, triglycerides, and glucose (n = 166). Given the reduced events-per-variable ratio (≈4 EPV), the omnibus model was fitted using Firth’s penalized logistic regression. No HLA marker reached statistical significance after BH correction (all p_adj_ ≥ 0.986; Table 4). HLA-DRB1*16 showed the smallest nominal *p*-value (OR = 0.49, 95% CI [0.16, 1.32], *p* = 0.165, p_adj_ = 0.986), followed by HLA-DRB1*14 (OR = 0.37, 95% CI [0.08, 1.60], *p* = 0.189, p_adj_ = 0.986).

Among covariates, BMI was strongly and significantly associated with NAFLD (OR = 1.22, 95% CI [1.12, 1.34], *p* < 0.001), and triglycerides were also significantly associated (OR = 1.48, 95% CI [1.04, 2.18], *p* = 0.031). Age (OR = 1.03, 95% CI [0.99, 1.08], *p* = 0.179) and glucose (OR = 1.14, 95% CI [0.80, 1.65], *p* = 0.461) were not significant.

### 3.3. Liver Fibrosis

Liver fibrosis was operationalized as a binary outcome based on liver stiffness measurement (LSM): participants with LSM ≥ 8 kPa were classified as having significant fibrosis (n = 19, 10.8%), whereas those with LSM < 8 kPa served as the reference group (n = 157, 89.2%).

There was a borderline difference in the prevalence of HLA-B*07 (*p* = 0.056) and a significant difference in HLA-DRB1*15 (*p* = 0.025) between patients with and without liver fibrosis (Table 5).

Given the low number of fibrosis events (n = 19, yielding ≈4 events per variable), all individual HLA models were fitted using Firth’s penalized logistic regression, adjusted for age, BMI, triglycerides, and glucose (Table 6). HLA-DRB1*15 showed the strongest association with fibrosis among all alleles tested (OR = 2.86, 95% CI [0.97, 8.04], *p* = 0.057), suggesting a near-threefold increase in odds among carriers; however, this did not reach conventional significance and was not significant after BH correction (p_adj_ = 0.624). HLA-B*07 also showed a trend-level association (OR = 2.33, 95% CI [0.79, 6.50], *p* = 0.121, p_adj_ = 0.642). Age was a borderline predictor across most models (*p*-values ranging from 0.049 to 0.064; see Table 6). No other HLA marker or covariate reached significance.

### 3.4. Liver Fibrosis Omnibus Model

An omnibus model including all eleven HLA markers was fitted using Firth’s penalized logistic regression, adjusted for age, BMI, triglycerides, and glucose (n = 166). No HLA marker reached statistical significance after BH correction (all p_adj_ ≥ 0.754; Table 7). HLA-DRB1*15 showed the largest effect size (OR = 2.64, 95% CI [0.79, 8.63], *p* = 0.111, p_adj_ = 0.754), consistent with the trend observed in the individual model. HLA-B*44 also showed an elevated but non-significant point estimate (OR = 1.97, 95% CI [0.48, 7.33], *p* = 0.328, p_adj_ = 0.754).

Among covariates, age showed a borderline association with fibrosis (OR = 1.06, 95% CI [1.00, 1.14], *p* = 0.064). BMI (OR = 1.00, *p* = 0.974), triglycerides (OR = 1.02, *p* = 0.960), and glucose (OR = 1.09, *p* = 0.700) were not significant in this model, in contrast to the NAFLD models where BMI and triglycerides were dominant predictors.

## 4. Discussion

The prevalence of NAFLD observed in this study was comparable to that reported in other studies and was associated with higher body weight, BMI, waist circumference (WC), hip circumference (HC), and triglycerides [14]. Triglycerides were a risk factor for NAFLD across all models, and BMI emerged as the strongest and most consistent predictor of NAFLD. Age showed a marginal, but not statistically significant, association with liver fibrosis. After adopting a parsimonious covariate structure, applying Firth’s penalized logistic regression where appropriate, and correcting for multiple testing using the Benjamini–Hochberg procedure, none of the examined HLA markers was significantly associated with NAFLD or fibrosis. The previously observed nominal association between HLA-DRB1*14 and reduced NAFLD risk was not reproduced in the revised analysis, consistent with its low carrier frequency and the instability of the original overparameterized specification. HLA-DRB1*15 showed the most consistent trend-level association with fibrosis across model specifications, but this did not survive FDR correction and should therefore be interpreted as hypothesis-generating.

NAFLD is characterized by triglyceride accumulation within hepatocytes [4]. One study reported that HLA-DR expression in monocytes at baseline varies with blood lipid levels and glycemic control in individuals with diabetes. Moreover, increased HLA-DR expression has been observed even in non-immune cells, for example, in atherosclerosis, suggesting more generalized immune activation in the context of metabolic pathology [15]. A large Chinese study of approximately 1.8 million healthy donors aged 18–50 years identified HLA-B*07, HLA-DRB1*07, and HLA-DRB1*12 as being associated with higher BMI, and HLA-C*03:02 as being associated with lower BMI [16]. A genetic analysis revealed an association between biological aging and fibrosis in young and middle-aged obese patients with MASLD [17]. In our cohort, patients with liver fibrosis were older than those without.

### 4.1. HLA, NAFLD and T Cell Involvement

In the present cohort, none of the tested HLA markers showed a statistically robust association with imaging-defined NAFLD or fibrosis after parsimonious modeling, penalized regression where appropriate, and correction for multiple testing. Accordingly, the immunological considerations below are presented as potential mechanistic context and hypotheses for future work rather than as inferences drawn from significant associations in our data.

Inflammation and metabolic syndrome are closely linked through T-cell-mediated mechanisms. In metabolic tissues, including adipose tissue, T cells may shift from immune regulation towards sustaining chronic low-grade inflammation, thereby worsening insulin resistance and promoting disease progression. Pro-inflammatory T-cell subsets (Th1, Th17) may predominate over anti-inflammatory counterparts (Tregs), and metabolic signaling pathways (mTOR, AMPK) are implicated in shaping these responses. This T-cell-driven inflammatory milieu contributes to insulin resistance, type 2 diabetes, NAFLD, and atherosclerosis, which are key features of metabolic syndrome [18]. HLA molecules are cell-surface proteins that enable T lymphocytes to recognize antigens. Class I HLA molecules (HLA-A, HLA-B, HLA-C) are expressed by virtually all nucleated cells and present peptides derived from intracellular proteins to CD8 T cells. In contrast, class II HLA molecules (HLA-DP, HLA-DQ, HLA-DR) are primarily expressed by professional antigen-presenting cells, such as B cells, dendritic cells, and macrophages, and present peptides generated from extracellular proteins that have been internalized and processed, to CD4 T cells. Importantly, HLA loci exhibit strong linkage disequilibrium across the MHC region, such that specific alleles are inherited together more often than expected by chance. This genetic architecture can complicate the attribution of disease associations to individual alleles, because observed effects may reflect correlated variation across linked loci rather than a single causal allele [19,20]. Cells of the innate immune system include Kupffer cells, monocytes, macrophages, hepatic dendritic cells, neutrophils, and natural killer (NK) cells, which bridge innate and adaptive immunity. The adaptive immune system comprises T cells (CD8, CD4—Th1, Th17, regulatory), B1 and B2 cells, and platelets. The interactions among these cellular subsets in NAFLD pathogenesis require further clarification [21]. Different T-cell subsets may have distinct effects on hepatic inflammation, fibrosis, and the progression from NASH to HCC; however, their roles in NAFLD are not fully understood [22]. A recent Mendelian randomization study that investigated immune cell phenotypes as potential causal factors in liver disease progression suggested that anti-inflammatory strategies targeting macrophage-specific receptors might reduce hepatic inflammation. The authors also emphasized the need for further work to define the functional traits, immunoregulatory networks, and molecular mechanisms underpinning these causal relationships [23]. Hepatocyte stress sensing is considered an early event in MASLD and involves γδ double-negative CD4−CD8 T cells that can produce pro-inflammatory mediators, including IL-17A. Subsequent recruitment of lymphocytes, such as Th17 cells, CD8 T cells, and B cells, may amplify inflammation, contributing to persistent inflammatory activity and fibrosis [24]. One study reported that the frequency of circulating NKG2D+ invariant NKT (iNKT) cells was negatively associated with hepatic steatosis, as assessed by CAP, suggesting that NK cells and iNKT cells may contribute to the regulation of hepatic steatosis and fibrosis in NAFLD [25]. These findings support the hypothesis that adaptive immunity contributes to NAFLD progression, potentially through antigen-driven hepatic inflammation and modulation of gut-liver immune interactions [26].

### 4.2. HLA Polymorphisms and NAFLD

Non-alcoholic fatty liver disease and autoimmune liver diseases are distinct entities, although they may coexist in some patients. Several studies have examined HLA associations in NAFLD, supporting a contribution of immune factors; however, the specific role of HLA-DRB1*14 in NAFLD susceptibility is less well established than that of other genetic determinants. HLA-DRB1*14 has been reported as an independent predictor of difficult-to-treat autoimmune liver disease, particularly in pediatric patients of Indian origin, and has been associated with poorer outcomes or treatment response [15]. In some Mediterranean, European, and Arab populations, HLA-DRB1*14 has been linked to a protective effect in RA, and a meta-analysis reported that this allele is less frequent in RA than in healthy individuals in these regions [15]. The frequency of HLA DRB1*14 in Europe ranges (5–10% combined), particularly when analyzing broader HLA-DRB1 allele groupings in European cohorts. Our results are consistent with previous studies on frequency. So far, this allele grouping has also been analyzed in terms of multiple sclerosis and achalasia. In our cohort, the frequency of HLA-DRB1∗14 was also lower than that of the other HLA-DRB1 alleles examined. In a study of 468 patients with chronic hepatitis B virus infection, carriers of HLA-DRB1*14:01:01 had significantly higher 6-year cumulative rates of hepatocellular carcinoma (HCC) than non-carriers [27].

Although not directly attributable to NAFLD, HLA genes are implicated in liver-related immune dysregulation. For example, HLA-DRB1*15 may influence immune-mediated liver injury and has been reported in patients with primary sclerosing cholangitis (PSC), underscoring the complex genetic architecture of liver disease [19,28]. In northern European populations, HLA-DRB1*15:01 has been reported to protect against AIH-1, AIH-2, and autoimmune sclerosing cholangitis (ASC) [19]. Mechanistically, this allele encodes an alanine at position 71 within the peptide-binding groove of the HLA-DR molecule, a site that can influence peptide presentation to T cells and, consequently, disease susceptibility. In a study of 138 NAFLD patients and controls, HLA-DRB1*15 and HLA-DRB1*16 differed markedly between individuals with steatosis and metabolic syndrome and the control group; notably, the difference for HLA-DRB1*16 remained highly significant even in the absence of metabolic syndrome [29].

A broader genetic contribution of the MHC region to liver disease has been described, and genome-wide association studies (GWAS) have implicated variants in HLA loci on chromosome 6p21 in NAFLD susceptibility (e.g., mapped loci including HLA-DRB1 and HLA-DQA1; variant ID rs502172) [5]. GWAS and related studies have identified HLA loci, including HLA-B, HLA-DRB1, and HLA-DQB1, as candidate regions for NAFLD risk, including in lean individuals, in whom metabolic risk factors may be less pronounced. For example, rs2076529 within the HLA region was associated with NAFLD in a Japanese cohort, and alleles such as HLA-B*54:01 were enriched among affected individuals, supporting a potential role for adaptive immunity in disease pathogenesis [6]. Similarly, high-resolution genotyping studies have reported allele-specific effects, with HLA-DQB1*06:04 conferring substantially increased risk (OR ≈ 7.3) and HLA-DQB1*03:02 appearing protective, consistent with immune-mediated contributions to hepatic injury [7]. These observations align with mechanistic models of NAFLD progression that emphasize chronic low-grade inflammation and immune activation. Because HLA molecules govern antigen presentation and T-cell responses, polymorphisms may influence the hepatic immune microenvironment and thereby contribute to steatohepatitis and fibrosis. Interactions between immunogenetic factors and lipid-metabolism genes, such as PNPLA3, may also contribute to inter-individual variability in disease severity and treatment response. Patatin-like phospholipase domain-containing protein 3 (PNPLA3) is a gene and protein involved in hepatic and adipose lipid metabolism and is implicated in the regulation of triglyceride storage and hydrolysis [30].

Other alleles, such as HLA-B*27 and HLA-A*31, have been associated with more severe histological features, including advanced fibrosis and high-grade steatosis, and an increased NAFLD risk has been reported for HLA-DRB1*07 [7]. In a study of 93 biopsy-proven NAFLD patients, HLA-DQB1*06:04 and HLA-DQB1*03:02 were observed at higher frequencies in NAFLD than in healthy controls [31]. Collectively, these findings support increasing evidence that adaptive immunity, mediated by specialized lymphocyte populations (T and B cells), contributes to NAFLD development and progression, likely through two broad pathways. First, antigen recognition within the liver can trigger activation of adaptive immune cells and sustain inflammatory responses; persistent inflammation may promote progression from steatosis to non-alcoholic steatohepatitis (NASH) and fibrosis. Second, adaptive immunity may contribute through modulation of gut–liver immune interactions [19]. Because the gut and liver are connected via the portal circulation, changes in the gut microbiome and/or increased intestinal permeability may allow microbial products to reach the liver and shape local immune responses, thereby influencing inflammation and disease progression. Future studies should characterize immune gene-expression changes and immune-cell infiltration patterns across NAFLD stages to further define these immunogenetic links.

Several findings approached, but did not reach, conventional thresholds for statistical significance, indicating that observed trends may reflect limited statistical power. The relatively small sample size is a likely contributor to these borderline findings. In addition, HLA genotyping was performed at low resolution using PCR-based methods.

Although this approach allows identification of broader allele groups, it does not reliably distinguish closely related alleles compared with high-resolution typing. As a result, potentially relevant sub-allelic differences may not have been captured in the present analysis.

### 4.3. Limitations

This study also has limitations related to residual confounding. Although we adjusted for key metabolic measures available in the dataset (anthropometrics, lipids, and glucose) and applied predefined exclusions for alcohol intake >20 g/day, we did not have sufficiently complete, standardized data to model additional factors such as HbA1c, hypertension status, statin exposure, detailed bDMARD/tsDMARD treatment classes, or long-term cumulative glucocorticoid burden. Therefore, unmeasured or imperfectly measured confounding cannot be excluded. Although detailed bDMARD/tsDMARD class exposure was not modeled in the present analysis, our prior CAP-based transient-elastography study in RA did not identify an association between MTX/biologic/targeted therapies and steatotic liver disease, suggesting that treatment class may be a weaker determinant of CAP-defined steatosis than metabolic factors in this setting [9]. Additional limitations include the cohort being predominantly female, which reflects the epidemiology of RA but may limit generalizability to male patients. Furthermore, the study did not include a non-RA control group; therefore, the findings address within-cohort associations rather than disease-specific excess risk relative to the general population. Lastly, because HLA allele frequencies differ markedly across populations, and our cohort predominantly reflected European ancestry allele distributions, the findings may not generalize to cohorts with different ancestral backgrounds or different prevalent alleles.

## 5. Conclusions

Comprehensive evaluation of HLA as potential contributors to NAFLD onset and progression may help clarify whether immunogenetic factors contribute meaningfully to steatotic liver disease in RA. In the present cohort, however, no robust association between the tested HLA markers and imaging-defined NAFLD or liver fibrosis was observed after use of parsimonious covariate adjustment, penalized regression in sparse-data settings, and correction for multiple testing. Metabolic factors—particularly BMI and triglycerides—were the dominant predictors of NAFLD. While a trend-level association of HLA-DRB1*15 with fibrosis was observed, it did not remain significant after FDR correction and should be regarded as hypothesis-generating. Larger studies with higher allele carrier counts and longitudinal follow-up will be required to determine whether any HLA variants are reliably associated with steatotic liver disease risk or fibrosis progression in RA.

## Figures and Tables

**Table 1:** Baseline Characteristics Stratified by NAFLD Status.

		NAFLD Status (CAP ≥ 275)		
Characteristic	Overall N = 176 ^1^	No N = 114 ^1^	Yes N = 62 ^1^	*p*-Value ^2^
Gender (Female)				>0.9
Female	152 (86.4%)	98 (86.0%)	54 (87.1%)	
Male	24 (13.6%)	16 (14.0%)	8 (12.9%)	
Age (years)	61.0 (53.2, 65.5)	60.4 (52.8, 65.0)	62.3 (56.3, 66.2)	0.065
Weight (kg)	73.0 (65.0, 84.5)	68.0 (64.0, 77.0)	82.5 (73.0, 91.0)	<0.001
BMI (kg/m^2^)	25.8 (23.5, 29.5)	24.5 (23.0, 26.6)	29.3 (26.5, 32.4)	<0.001
Waist circumference (cm)	92.0 (83.0, 103.5)	88.0 (81.0, 96.0)	103.0 (98.0, 112.0)	<0.001
Hip circumference (cm)	104.0 (98.0, 110.0)	102.0 (96.0, 106.0)	110.0 (105.0, 118.0)	<0.001
Total cholesterol (mmol/L)	5.7 ± 1.3	5.8 ± 1.3	5.6 ± 1.4	0.5
Triglycerides (mmol/L)	1.4 (0.9, 2.0)	1.2 (0.8, 1.9)	1.7 (1.1, 2.2)	0.002
Glucose (mmol/L)	5.1 (4.6, 5.7)	5.0 (4.6, 5.5)	5.2 (4.7, 5.9)	0.094
Currently on MTX	124 (70.5%)	75 (65.8%)	49 (79.0%)	0.084
Mean MTX dose (mg/week)	13.6 ± 4	12.3 + 3.8	15.9 + 4	0.34
MTX cumulative dose (mg)	960.0 (240.0, 3840.0)	930.0 (240.0, 3480.0)	960.0 (540.0, 4240.0)	0.3
HLA-A*02	101 (58.0%)	70 (62.5%)	31 (50.0%)	0.15
HLA-B*07	33 (18.9%)	20 (17.5%)	13 (21.3%)	0.5
HLA-B*08	40 (22.7%)	25 (21.9%)	15 (24.2%)	0.9
HLA-B*27	24 (13.7%)	17 (14.9%)	7 (11.5%)	0.6
HLA-B*44	31 (17.6%)	21 (18.4%)	10 (16.1%)	0.8
HLA-B*35	43 (24.4%)	26 (22.8%)	17 (27.4%)	0.6
HLA-B*51	34 (19.3%)	22 (19.3%)	12 (19.4%)	>0.9
HLA-DRB1*11	31 (17.8%)	19 (16.8%)	12 (19.7%)	0.7
HLA-DRB1*15	30 (17.2%)	21 (18.6%)	9 (14.8%)	0.7
HLA-DRB1*14	12 (6.9%)	7 (6.2%)	5 (8.2%)	0.8
HLA-DRB1*16	29 (16.7%)	22 (19.5%)	7 (11.5%)	0.2
Fibrosis (LSM ≥ 8 kPa)	19 (10.8%)	10 (8.8%)	9 (14.5%)	0.3

^1^ n (%); Median (Q1, Q3); Mean ± SD. ^2^ Fisher’s exact test; Wilcoxon rank sum test Continuous variables reported as mean ± SD if normally distributed (Shapiro–Wilk *p* ≥ 0.05 in both NAFLD groups), otherwise median (Q1, Q3). Categorical variables: n (%). *p*-values: Wilcoxon rank-sum (continuous), Fisher’s exact (categorical).

**Table 2:** Raw Carrier Frequencies: HLA Allele × NAFLD Status.

HLA Marker	No NAFLD Non-Carrier	No NAFLD Carrier	NAFLD Non-Carrier	NAFLD Carrier	Total N	Carrier Prevalence
HLA-A*02	42	70	31	31	174	58.0%
HLA-B*07	94	20	48	13	175	18.8%
HLA-B*08	89	25	47	15	176	22.7%
HLA-B*27	97	17	54	7	175	13.7%
HLA-B*44	93	21	52	10	176	17.6%
HLA-B*35	88	26	45	17	176	24.4%
HLA-B*51	92	22	50	12	176	19.3%
HLA-DRB1*11	94	19	49	12	174	17.8%
HLA-DRB1*15	92	21	52	9	174	17.2%
HLA-DRB1*14	106	7	56	5	174	6.9%
HLA-DRB1*16	91	22	54	7	174	16.7%

Note. Columns show counts of non-carriers and carriers within each NAFLD stratum (CAP ≥ 275 dB/m). HLA-DRB1*14 had the lowest carrier frequency (n = 12, 6.9%), with only 5 carriers in the NAFLD group and 7 in the non-NAFLD group. Minor variations in total N reflect missing data for specific alleles.

**Table 3:** Individual HLA Model Predicting NAFLD (Standard Logistic Regression).

HLA Marker	OR [95% CI]	*p*	p_adj_	n	BMI *p*	TR *p*
A*02	0.67 [0.32, 1.39]	0.281	0.632	170	<0.001	0.062
B*07	0.80 [0.31, 1.98]	0.642	0.883	171	<0.001	0.041
B*08	1.61 [0.68, 3.77]	0.273	0.632	172	<0.001	0.044
B*27	0.73 [0.24, 2.04]	0.567	0.883	171	<0.001	0.036
B*44	1.18 [0.42, 3.20]	0.746	0.912	172	<0.001	0.046
B*35	0.96 [0.42, 2.17]	0.927	0.927	172	<0.001	0.049
B*51	0.92 [0.35, 2.29]	0.862	0.927	172	<0.001	0.049
DRB1*11	1.29 [0.50, 3.22]	0.594	0.883	170	<0.001	0.053
DRB1*15	0.53 [0.19, 1.38]	0.208	0.632	170	<0.001	0.067
DRB1*14	0.44 [0.09, 1.91]	0.287	0.632	170	<0.001	0.043
DRB1*16	0.49 [0.17, 1.30]	0.170	0.632	170	<0.001	0.062

Note. OR = odds ratio; CI = confidence interval. Reference category: non-carriers. All models adjusted for age, BMI, triglycerides (TR), and glucose. p_adj_ = Benjamini–Hochberg adjusted *p*-value across 11 HLA markers. No HLA marker reached significance at α = 0.05 after correction.

**Table 4:** HLA Markers and NAFLD: Individual and Omnibus Models.

HLA	Individual OR [95% CI]	*p*	p_adj_	Omnibus OR [95% CI]	*p*	p_adj_
A*02	0.67 [0.32, 1.39]	0.281	0.632	0.83 [0.38, 1.82]	0.635	0.986
B*07	0.80 [0.31, 1.98]	0.642	0.883	0.99 [0.34, 2.74]	0.986	0.986
B*08	1.61 [0.68, 3.77]	0.273	0.632	1.45 [0.56, 3.69]	0.437	0.986
B*27	0.73 [0.24, 2.04]	0.567	0.883	0.82 [0.26, 2.37]	0.719	0.986
B*44	1.18 [0.42, 3.20]	0.746	0.912	1.40 [0.46, 4.20]	0.549	0.986
B*35	0.96 [0.42, 2.17]	0.927	0.927	0.97 [0.39, 2.37]	0.941	0.986
B*51	0.92 [0.35, 2.29]	0.862	0.927	1.03 [0.37, 2.78]	0.950	0.986
DRB1*11	1.29 [0.50, 3.22]	0.594	0.883	1.14 [0.44, 2.88]	0.780	0.986
DRB1*15	0.53 [0.19, 1.38]	0.208	0.632	0.60 [0.21, 1.61]	0.318	0.986
DRB1*14	0.44 [0.09, 1.91]	0.287	0.632	0.37 [0.08, 1.60]	0.189	0.986
DRB1*16	0.49 [0.17, 1.30]	0.170	0.632	0.49 [0.16, 1.32]	0.165	0.986

Note. OR = odds ratio; CI = confidence interval. Individual models: standard logistic regression (glm, binomial). Omnibus model: Firth’s penalized logistic regression. All models adjusted for age, BMI, triglycerides, and glucose. p_adj_ = Benjamini–Hochberg corrected across 11 HLA markers. No HLA marker reached significance at α = 0.05 after correction. Previously, HLA-DRB1*14 reached nominal significance (*p* = 0.047) in the original omnibus model, which included 10 covariates and used standard logistic regression; this finding did not survive the revised parsimonious specification or multiple-testing correction.

**Table 5:** Baseline Characteristics Stratified by Fibrosis Status.

		Fibrosis Status (LSM ≥ 8 kPa)		
Characteristic	Overall N = 176 ^1^	No N = 157 ^1^	Yes N = 19 ^1^	*p*-Value ^2^
Gender (Female)				0.3
Female	152 (86.4%)	137 (87.3%)	15 (78.9%)	
Male	24 (13.6%)	20 (12.7%)	4 (21.1%)	
Age (years)	61.0 (53.2, 65.5)	60.6 (52.8, 65.0)	66.2 (59.8, 68.4)	0.010
Weight (kg)	73.0 (65.0, 84.5)	73.0 (65.0, 82.0)	81.0 (66.0, 90.0)	0.070
BMI (kg/m^2^)	25.8 (23.5, 29.5)	25.7 (23.5, 29.3)	27.6 (23.8, 31.1)	0.2
Waist circumference (cm)	92.0 (83.0, 103.5)	92.0 (83.0, 103.0)	100.0 (82.0, 116.0)	0.2
Hip circumference (cm)	104.6 ± 10.7	104.3 ± 10.5	107.7 ± 12.2	0.2
Total cholesterol (mmol/L)	5.7 ± 1.3	5.8 ± 1.4	5.3 ± 1.2	0.2
Triglycerides (mmol/L)	1.4 (0.9, 2.0)	1.3 (0.9, 2.0)	1.5 (1.1, 2.1)	0.4
Glucose (mmol/L)	5.1 (4.6, 5.7)	5.1 (4.6, 5.6)	5.4 (4.6, 6.3)	0.2
Currently on MTX	124 (70.5%)	109 (69.4%)	15 (78.9%)	0.6
Mean MTX dose (mg/week)	13.6 ± 3.9	13.4 ± 4	15.2 ± 2	0.8
MTX cumulative dose (mg)	2455.7 ± 3456.3	2344.0 ± 3337.2	3366.6 ± 4306.6	0.2
HLA-A*02	101 (58.0%)	91 (58.3%)	10 (55.6%)	0.8
HLA-B*07	33 (18.9%)	26 (16.7%)	7 (36.8%)	0.056
HLA-B*08	40 (22.7%)	36 (22.9%)	4 (21.1%)	>0.9
HLA-B*27	24 (13.7%)	23 (14.7%)	1 (5.3%)	0.5
HLA-B*44	31 (17.6%)	27 (17.2%)	4 (21.1%)	0.7
HLA-B*35	43 (24.4%)	38 (24.2%)	5 (26.3%)	0.8
HLA-B*51	34 (19.3%)	30 (19.1%)	4 (21.1%)	0.8
HLA-DRB1*11	31 (17.8%)	30 (19.4%)	1 (5.3%)	0.2
HLA-DRB1*15	30 (17.2%)	23 (14.8%)	7 (36.8%)	0.025
HLA-DRB1*14	12 (6.9%)	11 (7.1%)	1 (5.3%)	>0.9
HLA-DRB1*16	29 (16.7%)	25 (16.1%)	4 (21.1%)	0.5
NAFLD (CAP ≥ 275 dB/m)	62 (35.2%)	53 (33.8%)	9 (47.4%)	0.3

Fibrosis defined as LSM ≥ 8 kPa. Continuous variables presented as mean ± SD if normally distributed (Shapiro–Wilk *p* ≥ 0.05 in both fibrosis groups), otherwise median (Q1, Q3). Categorical variables presented as n (%). *p*-values from Wilcoxon rank-sum test (continuous) or Fisher’s exact test (categorical). ^1^ n (%); Median (Q1, Q3); Mean ± SD. ^2^ Fisher’s exact test; Wilcoxon rank sum test.

**Table 6:** Individual HLA Models Predicting Fibrosis.

HLA Marker	OR [95% CI]	*p*	p_adj_	n	Age *p*	Age OR
A*02	1.13 [0.42, 3.20]	0.810	0.996	170	0.053	1.07
B*07	2.33 [0.79, 6.50]	0.121	0.642	171	0.051	1.06
B*08	1.10 [0.32, 3.26]	0.868	0.996	172	0.056	1.06
B*27	0.46 [0.05, 2.00]	0.332	0.913	171	0.062	1.06
B*44	1.46 [0.40, 4.47]	0.540	0.989	172	0.053	1.06
B*35	1.00 [0.32, 2.78]	0.996	0.996	172	0.056	1.06
B*51	1.04 [0.30, 3.08]	0.942	0.996	172	0.058	1.06
DRB1*11	0.35 [0.04, 1.50]	0.175	0.642	170	0.056	1.06
DRB1*15	2.86 [0.97, 8.04]	0.057	0.624	170	0.058	1.06
DRB1*14	0.66 [0.07, 3.20]	0.644	0.996	170	0.049	1.06
DRB1*16	1.51 [0.43, 4.51]	0.494	0.989	170	0.058	1.06

Note. OR = odds ratio; CI = confidence interval. All models fitted using Firth’s penalized logistic regression due to sparse outcome events (n = 19). Adjusted for age, BMI, triglycerides, and glucose. p_adj_ = Benjamini–Hochberg corrected across 11 HLA markers. No HLA marker reached significance at α = 0.05 after correction.

**Table 7:** HLA Markers and Fibrosis: Individual and Omnibus Models.

HLA	Individual OR [95% CI]	*p*	p_adj_	Omnibus OR [95% CI]	*p*	p_adj_
A*02	1.13 [0.42, 3.20]	0.810	0.996	1.04 [0.33, 3.53]	0.943	0.943
B*07	2.33 [0.79, 6.50]	0.121	0.642	1.62 [0.47, 5.27]	0.432	0.754
B*08	1.10 [0.32, 3.26]	0.868	0.996	1.30 [0.34, 4.42]	0.685	0.754
B*27	0.46 [0.05, 2.00]	0.332	0.913	0.54 [0.05, 2.76]	0.486	0.754
B*44	1.46 [0.40, 4.47]	0.540	0.989	1.97 [0.48, 7.33]	0.328	0.754
B*35	1.00 [0.32, 2.78]	0.996	0.996	1.39 [0.36, 5.06]	0.624	0.754
B*51	1.04 [0.30, 3.08]	0.942	0.996	1.35 [0.34, 4.82]	0.650	0.754
DRB1*11	0.35 [0.04, 1.50]	0.175	0.642	0.44 [0.05, 1.96]	0.308	0.754
DRB1*15	2.86 [0.97, 8.04]	0.057	0.624	2.64 [0.79, 8.63]	0.111	0.754
DRB1*14	0.66 [0.07, 3.20]	0.644	0.996	0.57 [0.05, 3.19]	0.555	0.754
DRB1*16	1.51 [0.43, 4.51]	0.494	0.989	1.42 [0.36, 4.90]	0.594	0.754

Note. OR = odds ratio; CI = confidence interval. All models fitted using Firth’s penalized logistic regression. Adjusted for age, BMI, triglycerides, and glucose. p_adj_ = Benjamini–Hochberg corrected across 11 HLA markers. HLA-DRB1*15 showed the strongest trend-level association (*p* < 0.10) in both the individual and omnibus specifications, but did not reach significance after correction.

## Data Availability

The data presented in this study are available on request from the corresponding author.

## Abbreviations

The following abbreviations are used in this manuscript:

ACR	American College of Rheumatology
EULAR	European League Against Rheumatism
ANA	Antinuclear antibody
BMI	Body mass index
CAP	Controlled attenuation parameter
GWAS	Genome-wide association study
LSM	Liver stiffness measurement
WC	Waist circumference
HC	Hip circumference
CHOL	Cholesterol
TR	Triglycerides
HLA	Human leukocyte antigen
MASLD	Metabolically–dysfunction–associated steatotic liver disease
MHC	Major histocompatibility complex
MTX	Methotrexate
MetS	Metabolic syndrome
NAFLD	Non-alcoholic fatty liver disease
NK	Natural killer
PCR-SSO	Polymerase chain reaction–sequence specific oligonucleotide
PCR-SSP	Polymerase chain reaction–sequence specific primer
PSC	Primary sclerosing cholangitis
RA	Rheumatoid arthritis
SNP	Single nucleotide polymorphosm
Γδ	Gamma-delta
NAsh	Non-alcoholic steatohepatitis
